# mSphere of Influence: the Dynamic Nature of the Nuclear Envelope during Mitosis of Malaria Parasites

**DOI:** 10.1128/mSphere.00815-20

**Published:** 2020-09-02

**Authors:** Sabrina Absalon

**Affiliations:** a Department of Pharmacology and Toxicology, Indiana University School of Medicine, Indianapolis, Indiana, USA

**Keywords:** *Plasmodium falciparum*, FIB-SEM, nuclear pore, chromatin packaging

## Abstract

Sabrina Absalon works in the field of cellular and molecular biology of Plasmodium falciparum, the most virulent parasite causing malaria in humans. In this mSphere of Influence article, she reflects on how the paper “3D nuclear architecture reveals coupled cell cycle dynamics of chromatin and nuclear pores in the malaria parasite Plasmodium falciparum” by Allon Weiner et al. (A. Weiner, N. Dahan-Pasternak, E. Shimoni, V. Shinder, et al., Cell Microbiol 13:967–977, 2011, https://doi.org/10.1111/j.1462-5822.2011.01592.x) triggered her aspiration to study the molecular mechanisms governing nuclear envelope assembly and integrity of P. falciparum throughout the intraerythrocytic development cycle.

## COMMENTARY

The driving force that motivates my scientific career is the desire to develop a greater understanding of the regulation of cell division machinery in eukaryotic cells. For decades, fission and budding yeasts have had a fantastic track record as model organisms that generate insights and unravel molecular mechanisms relevant across the eukaryotic domain. To date, it is still unknown what evolutionary factors have driven the diversity in the dynamic nature of the nuclear envelope (NE) seen in metazoan cells. As Theodosius Dobzhansky said, “Nothing in biology makes sense except in the light of evolution” (1). From a comparative biology viewpoint, several parasitologists like myself study the peculiar cell cycle of unicellular apicomplexan parasites to gain a functional understanding of mechanistic principles of eukaryotic cell division. Apicomplexans are obligate intracellular protozoans, which undergo distinct cell division modes to adapt to various hosts and cellular environments. Plasmodium falciparum, a member of the apicomplexan phylum, causes the most severe type of malaria. Clinically, malaria symptoms result from the replication of the parasite inside red blood cells (RBCs). The process of *Plasmodium* cell division differs from the classical cell cycle of its human host. In mammalian cells, which undergo open mitosis, the nuclear pore complexes (NPCs) disassemble along with the NE to leave room for the mitotic spindle to be formed in the cytosol. At the end of mitosis, the NE reassembles around the segregated chromatin into two new daughter cells. In contrast, P. falciparum replicates in red blood cells via schizogony wherein parasite nuclei undergo multiple asynchronous rounds of nuclear replication during which the NE remains intact, followed by segmentation, where the multinucleated cell completes a single round of specialized cytokinesis to construct daughter parasites around each nucleus (2, 3).

P. falciparum mitosis occurs with an intact NE, an ancient mechanism of eukaryotic cell division termed closed mitosis. Remarkably, the biological processes of NE formation and regulation remain mostly unknown and understudied in P. falciparum. Critically, no P. falciparum NE proteins have yet been identified aside from three nucleoporin proteins that compose NPCs (4). In this context, the work presented by Allon Weiner et al. (5) is a technical and conceptual milestone in our understanding of the dynamic nature of the NE during *Plasmodium* blood-stage replication. To examine the distribution of NPCs across the stages of P. falciparum cell cycle within the RBC, the authors applied a technology in cell biology named focused ion bean-scanning electron microscopy (FIB-SEM). This technique allows the user to slice (with the FIB) a plastic-embedded biological sample with nanoscale precision (down to 4 μm per slice) and to image (with the SEM) the freshly exposed surface to produce a two-dimensional (2D) image stack, which is then computationally converted to a high-resolution three-dimensional volume. The authors successfully employed this technique for the very first time on *Plasmodium* parasites, allowing them to unveil a striking correlation between the local distribution of nuclear pores and the chromatin organization as parasite development progresses.

The parasite starts the intraerythrocytic development cycle at the ring stage with few clustered NPCs (3 to 7 pores per nucleus), and the chromatin organization is diffuse within the cytoplasm. At the trophozoite stage, when the parasite remains mononucleated, the number of pores increases to 60 per nucleus as the parasite grows and begins replicating its DNA and organelles. After the first round of DNA replication is completed, the binucleated trophozoite harbors 29 to 38 NPCs per nucleus, the pores are uniformly spread around the NE, and small patches of heterochromatin are distributed throughout the nucleoplasm. Once the parasite has three or more nuclei, it is known as a schizont, and the nuclear organization changes dramatically. The number of NPCs per nucleus decreases to 6 to 16 in the mid-schizont stage down to 2 to 6 upon completion of cytokinesis. Throughout schizogony, NPCs cluster together adjacent to euchromatin and face the mother cell’s outer membrane. Unexpectedly upon cytokinesis completion, clusters of NPCs face toward the nascent daughter cell’s cytoplasm, suggesting a nuclear rotation during the transition from multinucleated cell to fully segmented parasites.

In addition to functioning as channels, NPCs play a role in gene regulation. Studies in yeast and *Drosophila* revealed that soluble nucleoporin proteins bind promoters of active genes at the nuclear periphery and enable active transcription (6, 7). In P. falciparum, the three-dimensional nuclear organization and positioning at the nuclear periphery are critical in transcriptional regulation, notably for virulence gene families (8). The mechanism governing the massive reorganization of the NPCs during *Plasmodium* replication remains elusive. The striking correlation between the dynamic change in chromatin organization and the NPC distribution during parasite development demonstrated by Weiner et al. suggest a central role of the NPCs in transcriptional regulation.

Throughout parasite replication, the nuclear envelope is actively remodeled, and dynamic changes in nuclear architecture are essential for parasite development. The work led by Allon Weiner et al. (5) triggered my scientific interest to study the mechanistic principles of NE assembly and integrity during the cell division of the human malaria parasite. From an evolutionary viewpoint, I wonder what advantage does asynchronous closed mitosis bring to *Plasmodium* species asexual replication? From a basic biology viewpoint, I wonder what molecular machinery orchestrates asynchronous mitosis in a multinucleated cell, and what cell cycle regulators direct multiple rounds of DNA replication with a single cytokinetic event?

Since the publication of this outstanding study, three research groups successfully applied the FIB-SEM technique to address gaps of knowledge in *Plasmodium* cell biology (9, 10). Notably, Rudlaff et al. demonstrated that P. falciparum parasite maintains nuclear division autonomy regardless of the cell cycle stage, a process previously reported synchronous upon cytokinesis (3).

Ultimately, elucidating the molecular mechanisms underlying P. falciparum nuclear division and NE biogenesis, two critical biological processes for parasite replication, will reveal druggable targets for the development of much needed new therapies against malaria as well as shed light on the evolutionary cell biology of eukaryotic cell division.

## References

[1] Dobzhansky T. 1973. Nothing in biology makes sense except in the light of evolution. Am Biol Teach 35:125–129. doi:10.2307/4444260.

[2] Gerald N, Mahajan B, Kumar S. 2011. Mitosis in the human malaria parasite Plasmodium falciparum. Eukaryot Cell 10:474–482. doi:10.1128/EC.00314-10. 21317311 PMC3127633

[3] Rudlaff RM, Kraemer S, Marshman J, Dvorin JD. 2020. Three-dimensional ultrastructure of Plasmodium falciparum throughout cytokinesis. PLoS Pathog 16:e1008587. doi:10.1371/journal.ppat.1008587. 32511279 PMC7302870

[4] Kehrer J, Kuss C, Andres-Pons A, Reustle A, Dahan N, Devos D, Kudryashev M, Beck M, Mair GR, Frischknecht F. 2018. Nuclear pore complex components in the malaria parasite Plasmodium berghei. Sci Rep 8:11249. doi:10.1038/s41598-018-29590-5. 30050042 PMC6062611

[5] Weiner A, Dahan-Pasternak N, Shimoni E, Shinder V, von Huth P, Elbaum M, Dzikowski R. 2011. 3D nuclear architecture reveals coupled cell cycle dynamics of chromatin and nuclear pores in the malaria parasite Plasmodium falciparum. Cell Microbiol 13:967–977. doi:10.1111/j.1462-5822.2011.01592.x. 21501361

[6] Schmid M, Arib G, Laemmli C, Nishikawa J, Durussel T, Laemmli UK. 2006. Nup-PI: the nucleopore-promoter interaction of genes in yeast. Mol Cell 21:379–391. doi:10.1016/j.molcel.2005.12.012. 16455493

[7] Mendjan S, Taipale M, Kind J, Holz H, Gebhardt P, Schelder M, Vermeulen M, Buscaino A, Duncan K, Mueller J, Wilm M, Stunnenberg HG, Saumweber H, Akhtar A. 2006. Nuclear pore components are involved in the transcriptional regulation of dosage compensation in Drosophila. Mol Cell 21:811–823. doi:10.1016/j.molcel.2006.02.007. 16543150

[8] Bunnik EM, Venkat A, Shao J, McGovern KE, Batugedara G, Worth D, Prudhomme J, Lapp SA, Andolina C, Ross LS, Lawres L, Brady D, Sinnis P, Nosten F, Fidock DA, Wilson EH, Tewari R, Galinski MR, Ben Mamoun C, Ay F, Le Roch KG. 2019. Comparative 3D genome organization in apicomplexan parasites. Proc Natl Acad Sci U S A 116:3183–3192. doi:10.1073/pnas.1810815116. 30723152 PMC6386730

[9] Medeiros LCS, De Souza W, Jiao C, Barrabin H, Miranda K. 2012. Visualizing the 3D architecture of multiple erythrocytes infected with Plasmodium at nanoscale by focused ion beam-scanning electron microscopy. PLoS One 7:e33445. doi:10.1371/journal.pone.0033445. 22432024 PMC3303842

[10] Rudlaff RM, Kraemer S, Streva VA, Dvorin JD. 2019. An essential contractile ring protein controls cell division in Plasmodium falciparum. Nat Commun 10:2181. doi:10.1038/s41467-019-10214-z. 31097714 PMC6522492

